# Identification of the Abundant Hydroxyproline-Rich Glycoproteins in the Root Walls of Wild-Type Arabidopsis, an *ext3* Mutant Line, and Its Phenotypic Revertant

**DOI:** 10.3390/plants4010085

**Published:** 2015-01-21

**Authors:** Yuning Chen, Dening Ye, Michael A. Held, Maura C. Cannon, Tui Ray, Prasenjit Saha, Alexandra N. Frye, Andrew J. Mort, Marcia J. Kieliszewski

**Affiliations:** 1 Department of Chemistry and Biochemistry, Ohio University, Athens, OH 45701, USA; E-Mails: Boxofjacky@gmail.com (D.Y.); held@ohio.edu (M.A.H.); af844006@gmail.com (A.N.F.); kielisze@helios.phy.ohiou.edu (M.J.K.); 2 Department of Biochemistry and Molecular Biology, University of Massachusetts, Amherst, MA 01003, USA; E-Mails: mcannon@biochem.umass.edu (M.C.C.); tuiray76@gmail.com (T.R.); sahap76@gmail.com (P.S.); 3 Biochemistry and Molecular Biology, Oklahoma State University, Stillwater, OK 74078, USA; E-Mail: Andrew.mort@okstate.edu

**Keywords:** *Arabidopsis* root, cell wall, hydroxyproline-rich glycoprotein, Extensin 3, LRX, PRP4, proteomics

## Abstract

Extensins are members of the cell wall hydroxyproline-rich glycoprotein (HRGP) superfamily that form covalently cross-linked networks in primary cell walls. A knockout mutation in *EXT3* (*AT1G21310*), the gene coding EXTENSIN 3 (EXT3) in *Arabidopsis* Landsberg *erecta* resulted in a lethal phenotype, although about 20% of the knockout plants have an apparently normal phenotype (ANP). In this study the root cell wall HRGP components of wild-type, ANP and the *ext3* mutant seedlings were characterized by peptide fractionation of trypsin digested anhydrous hydrogen fluoride deglycosylated wall residues and by sequencing using LC-MS/MS. Several HRGPs, including EXT3, were identified in the wild-type root walls but not in walls of the ANP and lethal mutant. Indeed the ANP walls and walls of mutants displaying the lethal phenotype possessed HRGPs, but the profiles suggest that changes in the amount and perhaps type may account for the corresponding phenotypes.

## 1. Introduction

Plants devote their extracellular space to a physically rigid cell wall comprised mainly of polysaccharides (cellulose, hemicellulose and pectin) and structural glycoproteins with a pectin-rich middle-lamella layer. Cell wall structural glycoproteins are rich in hydroxyproline (Hyp, O) and are thus named hydroxyproline-rich glycoproteins (HRGPs). HRGPs are ubiquitous components of the plant extracellular matrix and comprise up to ~10% (w/w) of the cell wall in dicots [1]. They create covalently crosslinked networks [2], generating negatively charged cushions at the membrane-wall interface [3], and thereby providing functions such as barriers against pathogen ingress [4,5], adhesives for pollen capture [6,7], primers for wall polysaccharide biosynthesis [8], and regulators of Ca^2+^ signaling [9,10].

HRGPs have characteristic repetitive peptide motifs that specify the extent and type of glycosylation on some, but not all, of the Hyp residues [11]. Three major types of HRGPs prevail in primary cell walls, namely the arabinogalactan proteins (AGPs), proline-rich proteins (PRPs), and extensins (EXTs). AGPs have short repetitive AO/SO/SOOAPAP motifs whereby most Hyp residues are heavily O-glycosylated with complex arabinogalactan polysaccharides. The AGP protein backbone accounts for less than 10% of the total molecule’s mass [12]. Many, but not all, AGPs have a GPI anchor at their C-terminus [13,14] and most AGPs react with β-glucosyl Yariv reagent to form a dark red precipitant, a common method for identifying AGPs [15]. Recently an Arabidopsis AGP was found to covalently interact with wall polysaccharide components rhamnogalacturonan I and arabinoxylan, indicating a structural role for AGPs in the cell wall architecture [8].

PRPs are lightly glycosylated HRGPs compared to AGPs, as sugar only makes up ~20% of the protein on a dry weight basis and they have repetitive peptide motifs such as POVXK, where X is often Tyr or Glu [16]. PRPs are expressed in various plant tissues and are tightly regulated during plant development and in response to environmental stimuli such as wounding [17].

EXTs are a type of HRGP that contribute to cell wall integrity by creating crosslinked networks during normal development [18], in response to wounding [5] and pathogen attack [4]. They are characterized by glycosylated Ser-(Hyp)_4_ motifs [19], whereby Ser residues are monogalactosylated and Hyp residues usually contain 1 to 4 oligoarabinosyl additions [20]. Another characteristic extensin motif is the Tyr-containing YXY motif that enables the covalent crosslinking of EXTs into the cell wall in the form of di-isodityrosine [21,22] and pulcherosine [23] presumably by wall bound peroxidases [24,25].

Earlier, Hall and Cannon identified a specific Arabidopsis EXT, designated RSH (Root Shoot Hypocotyl Defective) [26] and also known as EXT3, which is required for normal wall development. Loss of EXT3 due to a knock out mutation (the mutant designated “*rsh*”) was lethal due to abnormal and incomplete cell plate development [26,27]. Indeed mutant seedlings were visibly abnormal and died by three weeks of age.

This dramatic, readily observable difference between the mutant and wild-type phenotype provided a route to test EXT3 structure in wall assembly through the creation of altered forms of EXT3 used to rescue the mutant. However, this approach was complicated by the observation that some *rsh* homozygous lines (about 20%), although true mutants, as qPCR experiments found essentially no expression of the *EXT3* gene, possessed a wild-type phenotype, and were designated apparently normal phenotype (ANP) [28]. Judging by qRT-PCR and microarray experiments, we speculated that one or more proteins in ANP lines, including some predicted to be members of the HRGP family, recovered the wild-type phenotype in the absence EXT3.

We set out to characterize the different HRGPs contributing to the networks present in wild type (WT) Arabidopsis root walls, and root walls isolated from two ANP lines and from the *rsh* mutant displaying the lethal phenotype (MT) by characterizing the major HRGPs in these walls. Our approach involved tryptic peptide mapping of HF-deglycosylated wall to isolate the major peptides present followed by peptide sequence analysis by MS/MS.

Here we identified the major HRGP contributors to the WT, ANP, and MT root cell wall protein networks and in consistent with the results of Saha *et al.* [28], we found little evidence of EXT3 in ANP and MT roots while EXT3 was a major HRGP in the WT root.

## 2. Results

### 2.1. Arabidopsis Root Cell Wall Hyp/Protein Content

The HRGP contents in root cell walls of different Arabidopsis lines were first assessed by examining the amino acid compositions of WT, ANP and MT root cell walls. Hyp was found as the most abundant amino acid component of the WT Arabidopsis root cell wall matrix and comprised about 20% (mole) of the total amino acids (Table 1), thereby showing that HRGPs are the major components of the Arabidopsis root cell wall proteome. Hyp was also a major amino acid component of the WT root walls on a dry weight basis. Proteins comprised 7.3% (w/w) of the cell wall dry weight (Table 1) in the root walls, while the weight percentage of Hyp alone was 1.5% (w/w), making Hyp a 20.5% (w/w) component of wall proteins.

ANP and MT root walls shared similar amino acid compositions to WT walls with Hyp being the most abundant amino acid accounting for more than 17% (mole) of the total amino acids (Table 1). ANP and MT walls also showed a wall protein content similar to that of WT at around 7% (w/w). All walls had the same Hyp weight percentage of around 1.5%. Thus Hyp and the corresponding HRGPs were indeed major protein components in Arabidopsis root cell walls.

### 2.2. HF Deglycosylation of Root Cell Walls

To allow the release of Hyp-containing peptides from root cell walls for HRGP identification we then deglycosylated the walls with HF to remove wall polysaccharides. HF cleaves all neutral and acidic sugar linkages within 1 h at 0 °C but leaves peptide bonds intact [2], thus exposing the wall protein network and making it accessible to the later protease degradation. Here HF efficiently removed ~80% (w/w) of the cell wall dry weight from WT and ANP walls and 65% (w/w) of that in the case of MT walls (Table 1). Based on the weight percentage of protein and Hyp before and after HF, HF removed up to 40% (w/w) of the wall protein but little wall-bound Hyp, judging by the high content of Hyp (>90%, w/w) recovered from the remaining HF insoluble residue (HFI) (calculated from Table 1). Hyp remained the major amino acid that comprised more than 20% (mole) of the total amino acids in the root cell wall HFI of WT and ANP and about 15% (mole) in MT HFI (Table 1). The resistance of Hyp-containing wall component to HF deglycosylation indicates that HRGPs in the cell wall form an independent network from wall polysaccharides.

**Table 1:** Amino Acid Composition (mole %) of the Root Cell Wall (CW), Insoluble Cell Wall Pellet Remaining after Treatment with HF (HFI), Trypsin (TI HFI) and Pronase (PI HFI).

Amino Acid	WT	WT	WT	WT	ANP4	ANP4	ANP4	ANP4	ANP10	ANP10	ANP10	ANP10	MT	MT	MT
	CW	HFI	TI HFI	PI HFI	CW	HFI	TI HFI	PI HFI	CW	HFI	TI HFI	PI HFI	CW	HFI	TI HFI
Asp	6	1	2	1	6	6	4	4	6	4	1	1	7	5	5
Glu	6	3	2	2	5	4	3	3	6	3	2	2	7	2	4
**Hyp**	**20**	**25**	**27**	**30**	**17**	**22**	**20**	**22**	**18**	**29**	**22**	**28**	**17**	**15**	**19**
Ser	10	14	17	18	11	9	13	18	11	8	15	19	10	2	7
Gly	11	11	12	13	14	12	17	23	12	10	12	16	12	18	20
His	2	2	3	2	2	3	1	2	2	4	2	2	2	3	4
Arg	2	2	2	2	2	3	2	1	2	3	2	2	3	5	2
Thr	3	5	4	3	3	5	3	3	3	4	5	4	3	5	4
Ala	6	6	5	5	7	9	9	6	7	9	6	3	7	12	9
Pro	7	4	4	4	7	5	4	6	6	5	5	4	6	7	5
Tyr	4	2	2	2	3	2	3	2	3	2	1	2	3	2	1
Val	7	6	4	4	7	6	3	2	7	6	7	4	7	8	7
Ile	3	3	2	1	3	2	2	0	4	2	4	2	3	4	3
Leu	5	5	4	3	4	5	5	2	4	4	6	4	5	6	5
Phe	4	3	2	2	4	2	3	0	4	2	3	2	4	1	1
Lys	4	8	8	8	5	5	8	6	5	5	7	5	4	5	4
Total	100	100	100	100	100	100	100	100	100	100	100	100	100	100	100
% Hyp ^1^	1.5	6.9	5.2	4.5	1.1	3.9	2.1	1.1	1.3	6.3	4.3	4.1	1.1	2.5	3.8
% Protein ^1^	7.3	25.5	17.7	13.6	6.4	16.1	7	4.4	6.8	20.5	17.4	13.1	6.2	13.7	14.6
Hyp/ Protein ^2^	0.2	0.27	0.29	0.33	0.17	0.24	0.3	0.25	0.19	0.31	0.25	0.31	0.18	0.18	0.26
DW (mg) ^3^	50	10	8	7	38	10	7	6	51	10	8	7	4.6	1.6	0.75
% Hyp Removed ^4^		6.7	39.7	54.3		10.0	62.3	83.1		7.1	45.4	54.4		10.0	10.9
% Protein Removed ^4^		30.1	44.5	62.3		34.4	69.6	83.6		41.1	32.1	55.3		20.0	60.9

^1^ % Hyp or Protein: the weight percentage of Hyp and total protein; ^2^ Hyp/Protein: weight ratio of Hyp in total protein content; ^3^ DW (mg): the dry weight of cell wall used in HF deglycosylation and the remaining HFI before and after trypsin/pronase digestion; ^4^ % Hyp or Protein removed: the weight percentage of Hyp and protein removed after HF, trypsin or, pronase digestion calculated from Hyp/protein weight percentage of stepwise HFI, TI HFI and PI HFI.

### 2.3. Release of Hyp-Containing Material by Protease Degradation of Root Cell Wall HFI

Following HF deglycosylation of the root cell wall, WT, ANP, and MT HFI were sequentially treated with trypsin then pronase (WT and ANP only) to release wall peptides for protein identification. Exhaustive trypsin digestion of root wall HFI solubilized up to 60% (w/w) of the wall-bound proteins and more than 40% (w/w) of the Hyp-containing components from WT and ANP HFI, but only removed ~11% (w/w) Hyp from the MT HFI, although it removed 61% (w/w) of the MT HFI protein (Table 1). The trypsin insoluble HFI (TI HFI) of WT and ANP was further treated with pronase, which solubilized an additional ~20% (w/w) protein but a large portion of the wall bound Hyp (~50%, w/w) remained in the pellets that were resistant to proteolysis (Table 1). Hyp remained the most abundant amino acid in the protease insoluble residues, as indicated by the amino acid composition of the TI HFI and the pronase insoluble HFI (PI HFI, Table 1). Thus the HF insoluble protein network was somehow resistant to trypsin and pronase since only a portion of the Hyp-containing components in the HFI samples were released by protease degradation.

### 2.4. Peptide Fractionation from Trypsin Digested HFI of WT and ANP

**Figure 1:**
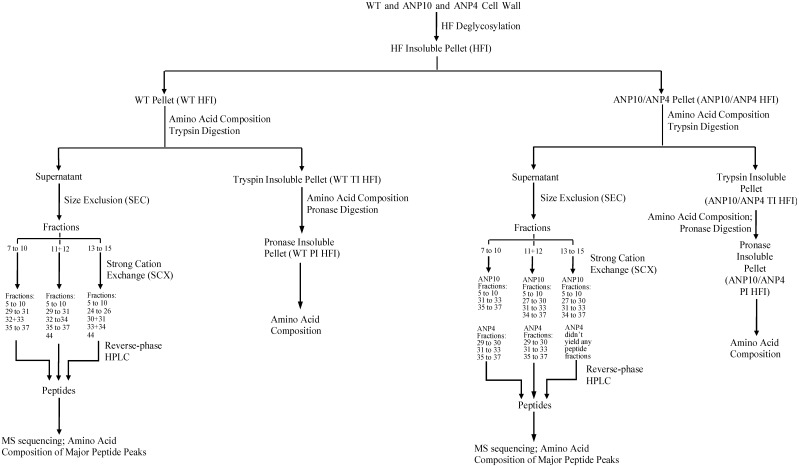
Flow chart outlining the isolation of WT, ANP10 and ANP4 root wall peptides. See Figure 2, Figure 3 and Figure 4 for the peptide maps from the SEC, SCX and reverse-phase HPLC columns.

Tryptic peptides from the WT and ANP walls were first fractionated to estimate the composition and relative abundance of specific HRGPs in these walls before applied for peptide sequencing by LC-MS/MS. Our approach combined size exclusion (SEC), strong cation exchange (SCX) and reverse phase chromatography, exploiting peptide properties to fractionate them (Figure 1).

**Figure 2:**
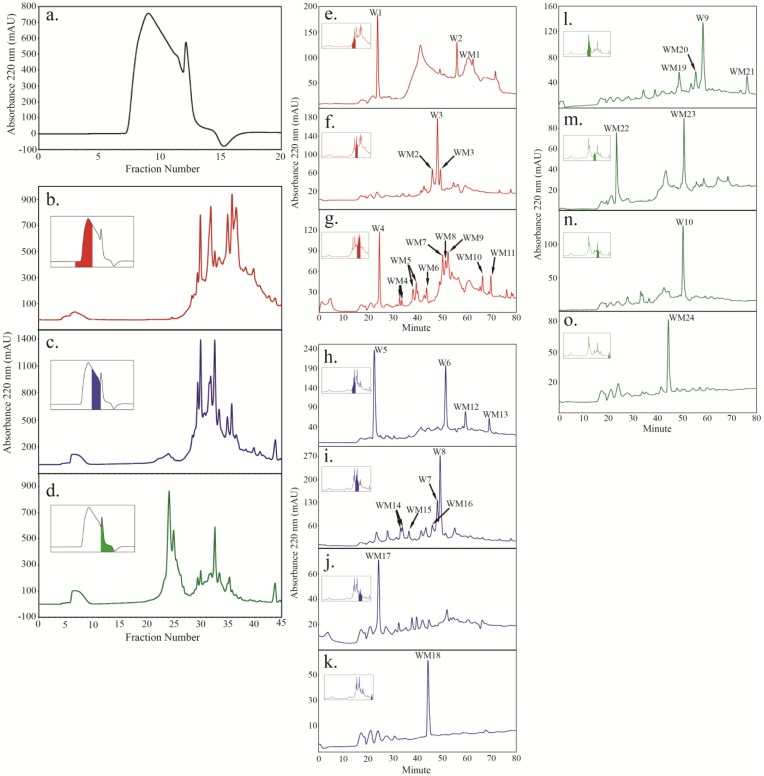
Peptides were isolated from trypsin digested WT HFI using a combination of size exclusion chromatography (SEC) (**a**), strong cation exchange chromatography (SCX) (**b**–**d**) and reverse phase chromatography (**e**–**o**), also summarized in Figure 1. Color-coded inserts in panels b to d identify the SEC fractions taken in turn for peptide mapping by SCX. Color-coded inserts in panels e through o identify the SCX fractions taken in turn for further peptide mapping by reverse phase chromatography. Major peptide peaks (A220 > 100) from WT HFI are labeled with “W” while minor peptides are labeled as “WM”.

Size fractionation of tryptic peptides liberated from WT HFI sample yielded three major fractions: a larger molecular weight major peak (fractions 7–10; Figure 2b inset), a broad shoulder peak (fractions 11–12; Figure 2c inset), and a sharp, smaller molecular weight peak (fractions 13–15; Figure 2d inset). Further separation of the SEC fractions 7–10 by SCX gave rise to five major peaks (Figure 2b) that, in turn, produced several peptide peaks after further fractionation by reverse-phase HPLC (Figure 2e–g). Four of these peaks were designated major peaks (W1, W2, W3, and W4) and several more as minor peaks (WM1-11). Similarly fractions 11–12 (Figure 2c inset) and fractions 13–15 (Figure 2d inset) from SEC were further fractionated by SCX and reverse-phase HPLC (Figure 2c,h,i,j and Figure 2d,L,m,n,o, respectively). Four major peptide peaks from the broad shoulder peak (W5, W6, W7 and W8) and two major peptide peaks from the small molecular weight SEC peak (W9 and W10) were identified.

Analysis of the Hyp content of the major WT wall peptide peaks indicated they all contained Hyp, with W3, W5, W6, and W10 being Hyp-rich, each containing more than 30% (mole) Hyp. W2 was relatively Hyp-poor and contained only 8% (mole) Hyp (Table S1).

**Figure 3:**
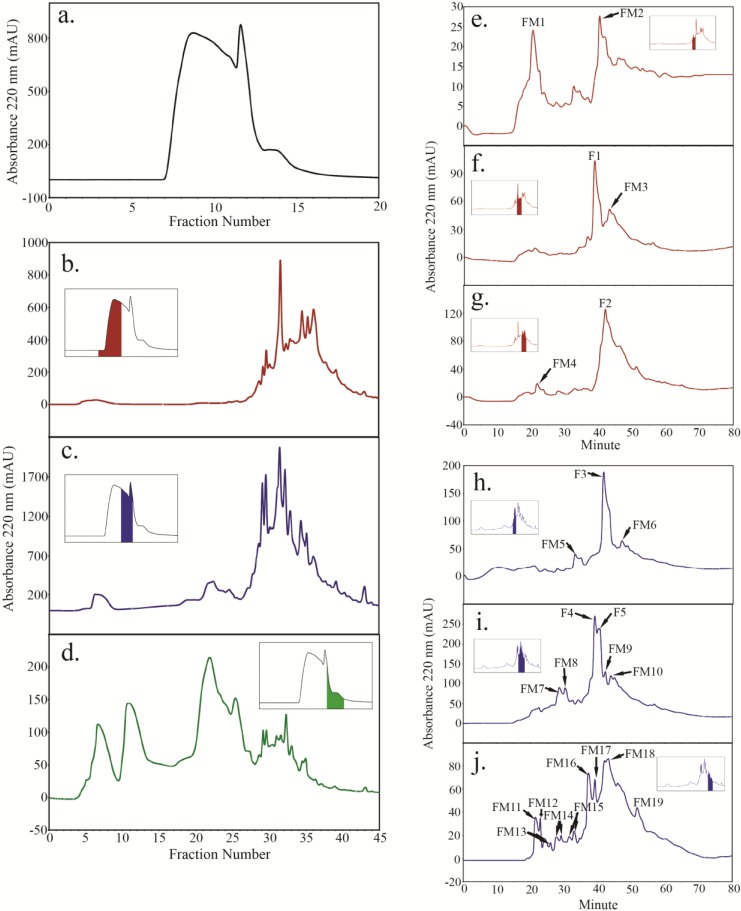
Peptides were isolated from trypsin digested ANP4 HFI using a combination of size exclusion chromatography (SEC) (**a**); strong cation exchange chromatography (SCX) (**b**–**d**) and reverse phase chromatography (**e**–**j**); also summarized in Figure 1. Color-coded inserts in panels (**b**) to (**d**) identify the SEC fractions taken in turn for further fractionation by SCX chromatography. Color-coded inserts in panels (**e**) to (**j**) identify the SCX fractions taken in turn for further peptide mapping by reverse phase chromatography. SCX fractions in (**d**) failed to yield any peptide peaks on further fractionation by reverse-phase HPLC. The major peptide peaks (A220 > 100) from the ANP4 HFI are labeled with “F” while minor peptides are labeled as “FM”.

**Figure 4:**
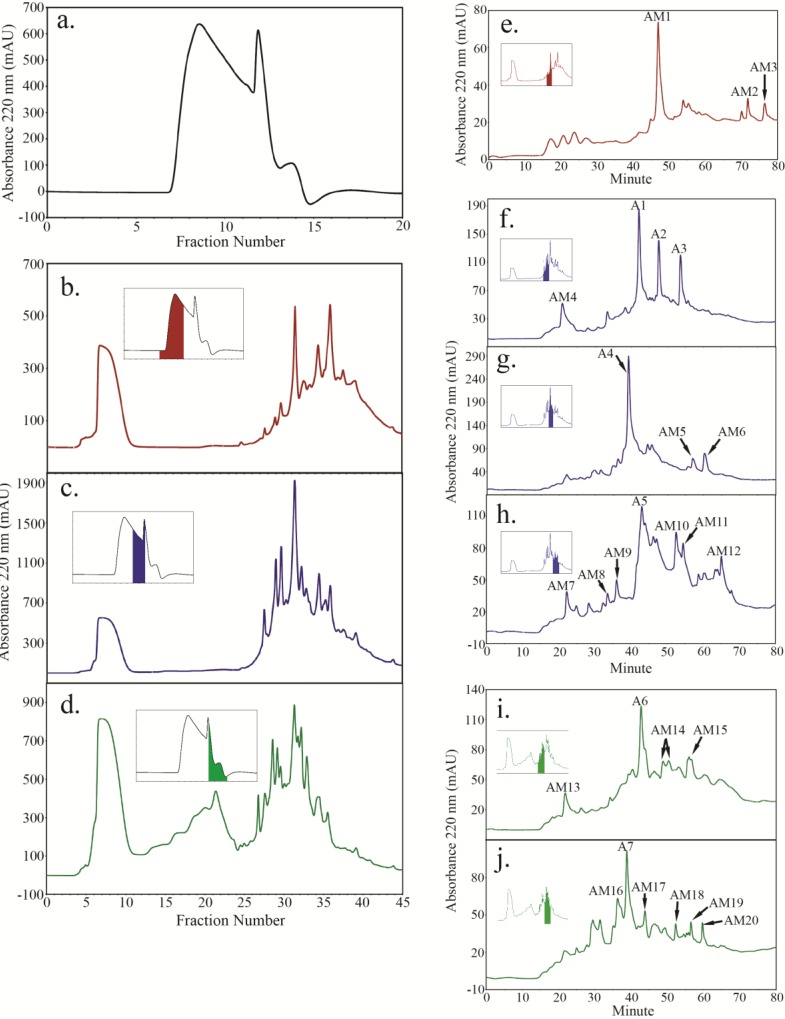
Peptides were isolated from trypsin digested ANP10 HFI using a combination of size exclusion chromatography (SEC) (**a**), strong cation exchange chromatography (SCX) (**b**–**d**) and reverse phase chromatography (**e**–**j**), also summarized in Figure 1. Color-coded inserts in panels b to d identify the SEC fractions taken in turn for further fractionation by SCX chromatography. Color-coded inserts in panels e to j identify the SCX fractions taken in turn for further peptide mapping by reverse phase chromatography. SCX fractions 35 to 37 in (**b**) and fractions 34 to 37 in (**d**) failed to yield any peptide peaks on further fractionation by reverse-phase HPLC. The major peptide peaks (A220 > 100) from the ANP10 HFI are labeled with “A” while minor peptides are labeled as “AM”.

Size fractionation of the trypsin soluble material from ANP4 and ANP10 HFI samples both resulted in profiles similar to that of WT (Figure 3a and Figure 4a). SEC fractions of ANP samples were grouped similarly to that of WT (fractions 7–10, 11–12 and 13–15, Figure 1) and fractionated by SCX and reverse-phase HPLC. For ANP4 HFI, SEC fractions 7–10 (Figure 3b inset) produced two major peptide peaks, F1 and F2 (Figure 3f,g), both of which were Hyp-rich, containing 32% (mole) and 42% (mole) Hyp, respectively (Table S1). SEC fractions 11–12 (Figure 3c inset) produced three major peptide peaks, F3, F4, and F5 (Figure 3h,i). F3 had 28% (mole) Hyp and F4 had 14% (mole) Hyp (Table S1). The Hyp content of F5 was not determined due to lack of material. SEC fractions 13–15 (Figure 3d inset) did not yield any peptide peaks judging by reverse-phase fractionation profile.

For ANP10 HFI, SEC fractions 7–10 (Figure 4b inset) did not yield any major peptide peaks but instead yielded three minor peptide peaks (AM1, AM2 and AM3, Figure 4e) after reverse-phase fractionation, despite the presence of several high UV-absorbing peaks evident during SCX fractionation (Figure 4b). SEC fractions 11–12 (Figure 4c inset) produced 5 major peptide peaks, A1 to A5 (Figure 4f,h) while SEC fractions 13–15 (Figure 4d inset) produced 2 major peptide peaks, A6 and A7 (Figure 4i,j) after reverse-phase fractionation. The Hyp content of the ANP10 major peptide peaks were low compared to those from WT and ANP4, with A1, A4, A5, and A6 containing ~17% (mole) Hyp (Table S1). A2 was Hyp-poor with 3% (mole) Hyp. No Hyp was detected in A3. The Hyp content of A7 was not determined due to lack of material.

The peptides released from the MT HFI were not fractionated as described above due to the low yields of MT walls. Instead, tryptic digests of the MT HFI were sequenced by LC-MS/MS without prior fractionation.

To sum up, we successfully isolated peptides from trypsin digested HFI of WT and ANP walls using a combination of chromatographic approaches and most of the major peptides were rich in Hyp, indicating the predominance of HRGPs in the root cell wall insoluble protein networks.

### 2.5. Peptide Sequencing and HRGP Identification

It has been suggested that the ANP phenotype may arise due to compensation by other HRGPs [27], which would likely result in changes in the protein composition of the HRGP network. Therefore, to compare the HRGP components of WT, ANP, and MT root walls, we performed LC-MS/MS peptide sequencing of peptides fractionated from WT HFI, ANP HFI, and MT HFI tryptic digests and identified HRGPs by their corresponding peptides. HRGPs that had two peptides passing the Scaffold 95% confidence filter or those having one peptide that passed the Scaffold 95% confidence filter but with a Mascot ion score larger than 40 were considered valid identifications.

Sequencing of fractionated peptides led to the identification of three EXTs (EXT1, EXT3, and EXT21), four AGPs (AGP30, AGP31, Fasciclin-like arabinogalactan proteins FLA7 and FLA15) and one PRP (PRP10) from WT HFI while a total of seven EXTs, including two classical EXTs (EXT1 and EXT17) and five chimeric EXTs (Leucine-rich Repeat/Extensin LRX1, LRX2, LRX3, LRX6 and HAE2), two AGPs (AGP31 and FLA16) and two PRPs (PRP4 and PRP10) were identified from ANP HFI (Table 2, see Figure S1 for full protein sequences of identified HRGPs).

Notably, uncertainties in the location of Hyp in some peptides were observed due to incomplete fragmentation of peptides. In those cases the total mass of a peptide suggested the existence of Pro hydroxylation on one or a few Pro residues, but the location of the Hyp residues can only be inferred from fragmented ion masses. For instance one AGP31 peptide AOVSOOAK(O/P)(O/P)VK(O/P)(O/P)VY(O/P)(O/P)TK had a mass of 2162.16, thus it contained 6 Hyp and 3 Pro residues. This peptide was identified in multiple locations on the reverse-phase HPLC fractionation peptide maps (Table 2) and showed various fragmentation patterns indicating variations in hydroxylation of the Pro residues, as reported earlier as well [29]. Similarly, this uncertainty of Hyp location was also observed in PRP4 peptides IEHPO(P/O)VOVYKOO(P/O)K and EVPO(P/O)VOVYK(P/O)(P/O)(P/O)K.

**Table 2:** HRGPs Identified in WT and ANP Cell Wall HFI.

Gene Accession #	Protein Identification (Plant Line of Origin)	Peptides Identified (Number of Occurrence in Protein Sequence; Peak of Peptide Identification) ^1^	Peptide Mascot Ion Score ^2^	Signal Peptide ^3^	MM (kDa) ^4^	pI ^4^
**EXTs**						
AT1G76930	EXT1 (**WT**, **ANP4**, **ANP10**)	YYSOOOVYK (**3**; W2, W6, W8, W10, WM3, WM13; FM16; A2)		1–24	32.9	10.1
		HYSOOOVYK (**9**; WM6; A5), SOOOOVYK (**1**; FM8)				
		SOOOOVK (**8**; W1, W2, W5, WM22; AM3)				
AT1G21310	EXT3, RSH (**WT**)	HYSOOOVYHSOOOOK (**13**; WM6), HYTOOVK (**1**; WM4),		1–28	49.2	10.3
		SOOOOVK (**13**; W1, W2, W5, WM22)				
AT3G54580	EXT17 (**ANP4**)	SOOOOYYSOSOK (**15**; FM8)	54.9	1–22	105.7	9.5
AT2G43150	EXT21^5^ (**WT**)	SOOOOYYYHSOOOOVK (**6**; W8, W10)	54.3	1–27	23.4	9.6
		SOOOOVK (**10**; W1, W2, W5, WM22)				
AT1G12040	LRX1 (**ANP4**, **ANP10**)	LQGPLPSSVGNMK (**1**; F3)	42.3	1–26	81	8.2
		LLYELDLSNNR (**1**; AM4)	44.5			
AT1G62440	LRX2 (**ANP4**, **ANP10**)	SLEQLNVANNR (**1**; FM4, FM12, FM13)	71.4		89.8	4.8
		LSGPLPSSIGNMK (**1**; A2)	59.4			
AT4G13340	LRX3 (**ANP10**)	FNEFEGTVPK (**1**; AM5, AM13, AM15, AM20)	65.9	1–20	82.2	7
AT3G22800	LRX6 (**ANP4**)	SLEQLNIAHNK (**1**; FM6)	55.7	1–28	52.3	6.2
AT3G50580	HAE2 (**ANP10**)	SOOOOTOK (**1**; AM3, AM12)	41.7	1–24	27.2	9.1
**AGPs**						
AT2G33790	AGP30 (**WT**)	LPAYPOAK (**1**; W2), TLVAVR (**1**; WM3), VSSLHDGGK (**1**; WM4)		1–25	25.8	10.6
AT1G28290	AGP31 (**WT**, **ANP4**, **ANP10**)	SLVAVR (**1**; W8, W10, WM3), SOVKPOVK (**1**; WM4, WM14),		1–25	38.4	10.8
		NITAETTTDK (**1**; FM8),				
		AOVSOOAK(O/P)(O/P)VK(O/P)(O/P)VY(O/P)(O/P)TK^7^				
		(**1**; W3, W4, W7, W8, W10, WM3, WM18, WM19, WM22; F1, F4, FM4, FM16; A5, AM1), LFGGDVGAELKPEK				
		(**1**; FM7, FM8, FM18; AM10, AM11, AM18)				
AT2G04780	FLA7 (**WT**)	FTDVSGTVR (**1**; W10, WM3)	61.4	1–22	26.8	6.5
AT3G52370	FLA15 (**WT**)	HHFNGEAQVK (**1**; WM24)	42.3	1–20	48.1	6.7	
AT2G35860	FLA16 (**ANP4**)	EETOATEIKPAAOVVK (**1**; F1, FM4, FM13)	55.4	1–23	49.1	6.8	
**PRPs**							
AT4G38770	PRP4 (**ANP4**)	EVPO(P/O)VOVYK(P/O)(P/O)(P/O)K^7^ (**1**; F1),		1–29	49.1	10.3	
		IEHPO(P/O)VOVYKOO(P/O)K^7^ (**1**; F2, FM18)					
AT5G09530	PRP10 (**WT**, **ANP4**, **ANP10**)	FPENSKPEVPK (**1**; WM8, WM9; F2, FM18; A5)	64.8 (in WT)	1–35 ^6^	41.6	5.9	
		VPEIPKPEETK (**1**; FM4, FM11, FM12) , MPEIQKPELPK					
		(**1**; FM4, FM12, FM13), LPDIPK (**1**; FM16),					
		LPEVPK (**1**; FM16), LPEFPKPELPK (**1**; AM4, AM18),					
		MPEIPKPELPK (1; AM20)					

^1^ Only peptides that pass the 95% Scaffold confidence filter are shown; ^2^ The Mascot ion score are listed for those HRGPs that only have one peptide that pass the 95% Scaffold confidence filter but with score > 40; ^3^ Signal peptides were predicted by SignalP (www.cbs.dtu.dk/services/SignalP); ^4^ Predicted protein molecular weight (MM) and isoelectric point (pI) values were obtained from TAIR (www.arabidopsis.org); ^5^ EXT21 shares one peptide (SOOOOVK) with EXT1 and EXT3, however the Mascot ion score of its unique peptide SOOOOYYYHSOOOOVK is larger than 40, thus validates the existence of EXT21; ^6^ For PRP10, SignalP prediction indicated the absence of a signal peptide while the cellular localization predicted by TargetP (http://www.cbs.dtu.dk/services/TargetP/) indicated a secretory pathway localization and thus the possession of a signal peptide. The signal peptide sequence and the putative cleavage site were determined according to the S-score (signal sequence score) and C-score (cleavage score) by SignalP; ^7^ The hydroxylation of some of the Pro residues in some AGP31 and PRP4 peptides could not be precisely determined, thus both possible sequences (either Pro-Hyp or Hyp-Pro) are indicated as P/O.

The peptides from the trypsin digested MT HFI were not fractionated by multiple chromatographic steps due to the low yield of the HFI material. Instead, the peptides from the trypsin digested MT HFI were directly sequenced by LC-MS/MS. This approach allowed the determination of protein relative abundance via the “total spectra count”, with higher spectra count value corresponding to higher protein abundance (Table 3). This feature did not apply to the peptide mapping approach since every peptide peak was sequenced individually thus the relative abundance of proteins identified by LC-MS/MS referred to the value in that particular peak. A total of 11 HRGPs were identified in MT walls including 8 EXTs, 2 AGPs and 1 PRP (Table 3). LRX2, LRX3 and LRX4 were the most abundant HRGPs identified in MT with high total spectra counts while PAG2, PRP3 and EXT3 were minor components of the MT wall judging by their low total spectra counts (Table 3). Notably, only a trace of EXT3 was identified with a total spectra count of 1 by peptide HYSOOOVYHSOOOOK that occurs 13 times in the protein. The low peptide abundance of such a major repetitive unit in EXT3 was consistent with the result of Saha *et al.* [28] who found EXT3 expression in the mutant was 0.06 times of that in WT [28]. Thus our result confirmed the virtual absence of EXT3 in MT walls.

In conclusion, here we identified several HRGP members in the root cell walls of WT, ANP and MT and in the meantime confirmed the absence of EXT3 in ANP and MT.

### 2.6. Comparison of HRGP Networks

**Figure 5:**
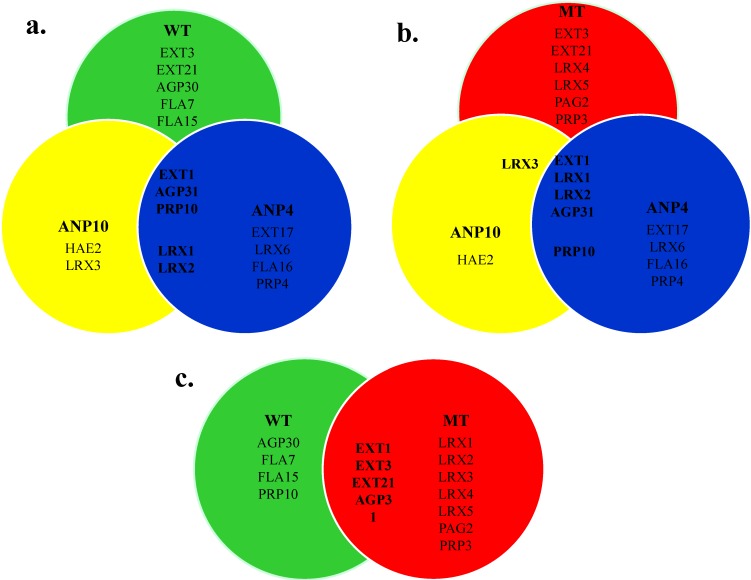
Comparison of members in HRGP networks of (**a**) WT and ANPs; (**b**) MT and ANPs and (**c**) WT and MT identified by proteomics approach.

Finally, the HRGP members in the root cell wall of different Arabidopsis lines identified by this proteomics approach were compared. WT Arabidopsis and *ext3* knockout lines (ANP and MT) showed somewhat different HRGP networks but the differences did not allow a diagnosis for the ANP phenotype. For WT and ANP plants that display a normal phenotype, EXT1, AGP31 and PRP10 are in common while the two ANP lines have two chimeric LRX EXTs, LRX1 and LRX2, in common (Figure 5a). Notably, despite the absence of the LRX proteins in WT, ANP4 HRGP network also differed from that of WT with the presence of EXT17 and PRP4 (Figure 5a) that could potentially contribute to the wild-type phenotype in ANP4.

The MT and ANP plants both lack EXT3 and despite the profound phenotypic differences, they both have EXT1, LRX1, LRX2 and AGP31 (Figure 5b). Here again worth noticing is that the presence of EXT17, LRX6, PRP4, and PRP10 in ANP4 but not in MT. These HRGPs could potentially result in the rescue of MT to restore the wild-type phenotype (Figure 5b). WT and MT plants have the classical EXTs, *i.e.*, EXT1, EXT3 and EXT21, as well as AGP31 in common, even though EXT3 is a minor component in the MT walls (Figure 5c).

### 2.7. Potential Candidate HRGPs for the Arising of ANP

Previously Saha *et al.* [28] explored the arising of the ANP using microarray analysis and qRT-PCR. Several HRGPs were proposed to be responsible for the ANP phenotype based on their elevated gene expression levels (Table 6 in [28]). In the current study we aimed to obtain biochemical evidence to address the ANP phenotype issue and in the meantime, support the findings by Saha *et al.* [28].

Here we indeed identified several HRGPs in the ANP walls (Table 2). However, while the proteomics analysis confirmed their existence, this data alone is not sufficient to make an assessment on their involvement in rescuing of the *ext3* knockout mutant. To provide more evidence, we compared the expression of genes coding for those ANP HRGPs identified by proteomics with their expression in WT and MT roots (Table 4) to identify potential candidates for the ANP phenotype.

In ANP4, the gene expression levels of most of the proteomics-identified HRPGs were slightly down regulated compared to WT, except for *PRP4*, which showed a more than 2-fold up-regulation. Notably, compared to MT, the expression level of three ANP4 HRGPs, *LRX1*, *PRP4* and *PRP10* showed more than 2-fold up regulation. Meanwhile, when compared to WT, the expression level of *LRX1*, *PRP4* and *PRP10* in MT showed a more than 2-fold down regulation [28]. This implies that ANP4 has increased expression of *LRX1*, *PRP4* and *PRP10* genes almost to the WT level. The peptide isolation reported here from the ANP4 wall confirmed that these proteins were indeed present and might possibly be applied by ANP4 to restore the normal phenotype. Similarly in the ANP10 line, LRX1, LRX2 and PRP10 were also candidates for the rescue (Table 4).

**Table 3:** HRGPs Identified in MT Cell Wall HFI.

Gene Accession #	Protein Identification	Peptides Identified ^1^ (Number of Occurrence in Protein Sequence)	Peptide Mascot Ion Score ^2^	Total Spectra Count ^3^	Signal Peptide ^4^	MM (kDa) ^5^	pI ^5^
**EXTs**							
AT1G76930	EXT1	HYSOOOVYK (**9**), YYSOOOVYK (**3**)		6	1–24	32.9	10.1
AT1G21310	EXT3, RSH	HYSOOOVYHSOOOOK (**13**)	46.6	1	1–28	49.2	10.3
AT2G43150	EXT21	SOOOOYYYHSOOOOVK (**6**)	54.4	3	1–27	23.4	9.6
AT1G12040	LRX1	KVTVFDITSNR (**1**), VTVFDITSNR (**1**), VVLSLPSLK (**1**)		5	1–26	81	8.2
AT1G62440	LRX2	ELGLLTDLALFHLNSNR (**1**), FPNVVLSLPSLK (**1**), SLEQLNVANNR (**1**)		10		89.8	4.8
AT4G13340	LRX3	DLDAIFINHNR (**1**), FNEFEGTVPK (**1**), FPTVVLQLPSLK (**1**)		11	1–20	82.2	7
		FRFELPENFGDSPVSVIVLANNR (**1**)					
AT3G24480	LRX4	FPTVVLHLPSLK (**1**), SAYIALQAWK (**1**)		11	1–25	54.7	6.9
AT4G18670	LRX5	FAGIFPTVVLQLPSLK (**1**), FNEFEGPVPR (**1**)		9	1–31 ^6^	90.8	6.7
**AGPs**							
AT1G28290	AGP31	LFGGDVGAELKPEK (**1**), NGYFLLLAPK (**1**), TVTNFGFR (**1**)		5	1–25	38.4	10.8
AT2G25060	PAG2	LSLVVISPR (**1**)	44.7	1	1–28	19.5	7.4
**PRPs**							
AT3G62680	PRP3	GLTGVPLALYGYR (**1**), SNTEVVIYSNPTDSK (**1**)		2	1–22	34.4	9.7

^1^ Only peptides that pass the 95% Scaffold confidence filter are shown; ^2^ The Mascot ion score are listed for those HRGPs that only have one peptide that pass the 95% Scaffold confidence filter but with score>40.; ^3^ Total spectra count indicates the relative abundance of protein in the sample; ^4^ Signal peptides were predicted by SignalP; ^5^ Predicted protein molecular weight (MM) and isoelectric point (pI) values were obtained from TAIR; ^6^ For LRX5, SignalP prediction indicated the absence of a signal peptide while the cellular localization predicted by TargetP indicated a secretory pathway localization and thus the possession of a signal peptide. The signal peptide sequence and the putative cleavage site were determined according to the S-score and C-score by SignalP.

**Table 4:** Gene Expression Patterns of Proteomics Identified ANP HRGPs using qRT-PCR: A Comparing of WT and *rsh* Mutant and ANP Revertants.

	Protein (Accession #)	Fold Change (Log_2_) ANP *vs.* WT ^1^	Fold Change (Log_2_) ANP *vs.* MT^1^
ANP4	EXTs:		
	AtEXT1 (AT1G76930)	0.22; (0.72) ^2^	−2.50; (0.00)
	AtEXT17 (AT3G54580)	−0.94; (0.13)	1.03; (0.20)
	LRX1 (AT1G12040)	−0.45; (0.29)	5.35; (0.02)
	LRX2 (AT1G62440)	−0.85; (0.05)	−0.66; (0.05)
	LRX6 (AT3G22800)	−0.50; (0.30)	−0.95; (0.05)
	AGPs:		
	AGP31 (AT1G28290)	n/d ^3^	n/d
	FLA16 (AT2G35860)	n/d	n/d
	PRPs:		
	PRP4 (AT4G38770)	1.20; (0.00)	2.25; (0.00)
	PRP10 (AT5G09530)	−0.55; (0.05)	3.30; (0.01)
ANP10	EXTs:		
	AtEXT1 (AT1G76930)	0.40; (0.04)	−1.69; (0.001)
	LRX1 (AT1G12040)	−0.07; (0.86)	6.90; (0.0001)
	LRX2 (AT1G62440)	−0.07; (0.79)	0.89; (0.01)
	LRX3 (AT4G13340)	0.64; (0.02)	−2.27; (0.0003)
	HAE2 (AT3G50580)	3.33; (0.02)	0.14; (0.77)
	AGPs:		
	AGP31 (AT1G28290)	n/d	n/d
	PRPs:		
	PRP10 (AT5G09530)	−1.33; (0.00)	3.7; (7.9 × 10^−6^)

^1^ The culture generation compared: ANP4/10-F3 *vs.* WT-F2, ANP4-F2 vs MT-F1 and ANP10-F3 *vs.* MT-F1; ^2^ Numbers in parenthesis: significance value, *p* ≤ 0.05 are considered statistically significant; ^3^ n/d: not determined.

## 3. Discussion

### 3.1. Structural Protein Network in Arabidopsis Root Cell Wall

HRGPs are major protein components in the dicot plant and cell culture walls as suggested by their high Hyp contents (>10 mole% of total amino acids) [30,31,32,33]. As a model dicot, Arabidopsis is of no exception as the amino acid composition of WT Arabidopsis root cell walls showed that it was indeed a Hyp-rich matrix (Table 1).

Cell wall structural proteins have long been proposed to form a covalently linked network in the wall [34]. This network appears to be independent from the other cell wall polysaccharide networks since it is preserved after HF treatment, which cleaves all glycosidic linkages in tomato suspension culture cell walls [2]. A previous study of the legumes root cell wall EXT components further confirmed this notion where HF deglycosylation of the cell wall removed two-thirds of the wall dry weight mass, about 29% (w/w) of the wall protein, but less than 3% (w/w) of the wall-bound Hyp [35]. Here we report a similar observation with Arabidopsis root cell walls that more than 90% (w/w) of the Hyp remained after HF deglycosylation whereas more than 60% of the cell wall dry weight and up to 40% (w/w) of the wall protein was removed (Table 1), indicating the insolubilized Hyp-rich protein network was firmly held in Arabidopsis root cell wall, most likely through covalent interactions.

### 3.2. Protease Degradation of HFI

The wall pellets after HF deglycosylation contained the remaining wall protein components that accounted for up to 25% of the WT wall dry weight while the weight percentage of protein in ANP and MT HF insoluble pellets were close to or less than 20% (Table 1). A large portion of the pellet weight was comprised of “unknown substances” that were neither protein nor sugar considering the high efficiency of HF deglycosylation [2]. The presence of such “unknown substances” in the HF insoluble wall was reported earlier for tomato suspension culture cells [2], legume root hairs [36], legume root and root nodules [34]. The nature of these substances has yet to be determined, but secondary cell wall substances such as lignin from root xylem vessels [37] and suberin from cuticles [38,39] are possibilities. Under the conditions of HF treatment used here the phenolic linkages of lignin would not have been cleaved [40] nor would the ester linkages between the fatty acid components of cutin, which are identical to those of suberin [41,42].

Hyp remained the most abundant amino acid in the protease insoluble residues and indeed protease digestion left the WT and MT pellets enriched in Hyp judging by the increase in the Hyp/protein ratio (Table 1). This observation suggested that the Hyp containing portion of the protein network is relatively more resilient to proteases. The associations between wall proteins and the “unknown substances” in the HF insoluble wall residues could account for the incomplete removal of Hyp-containing peptides by proteases. A significant portion of the wall protein is likely embedded or crosslinked to the network of the “unknown substances” [43,44,45] and therefore inaccessible to the proteases.

### 3.3. EXT3 in Root Cell Wall

Apart from EXT3, ANP and MT cell walls showed similar protein and Hyp contents to WT (Table 1). Therefore EXT3 is either a minor but crucial component of the wall as the knock-out of *ext3* does not affect the total protein content or in the absence of EXT3 other Hyp containing proteins are present. Although the absolute amount of EXT3 in these different Arabidopsis lines has not been determined, Saha *et al*. [28] showed in previous work that many HRGP genes have altered expression (some up and others down) in the ANP and MT plants compared to WT, while there was no significant difference in the total HRGP levels between WT, ANP and MT plants, supporting the latter notion.

Also surprising, is the fact that the abundant (13 repeats in EXT3) EXT3 peptide HYSOOOVYHSOOOOK was only found in a minor peptide fraction of WT; this may be because EXT3 is a minor component of the HFI or because the peptide remained with the trypsin insoluble wall pellet due to extensive intermolecular cross-lining. The latter possibility seems likely in light of the abundance of cross-linking sites on EXT3 molecule (see below) and as shown in earlier work with Arabidopsis cell suspension cultures that identified EXT3 as the major salt-elutable extensin present in growing cells [46].

### 3.4. Cross-linking of HRGPs into the Cell Wall

The mechanism for the formation of the covalently cross-linked wall HRGPs network is largely unknown. Tyr residues, especially those involved in the YXY motif, have been shown to form intramolecular isodityrosine (Idt) cross-links [47,48] that lead to insolubilization by intermolecular cross-linking to produce di-isodityrosine (Di-Idt) [21,22] and pulcherosine [23]. Another putative cross-linking motif is the VYK motif in HRGPs, as the monomeric P1 type tomato extensin (contains majorly VYK motif with only small amount of Idt) [49,50] cross-linked efficiently by a wall bound peroxidase [24]. Classical EXTs usually contain multiple Idts and/or VYK motifs that are candidates for pulcherosine formation.

For the classical EXTs identified in HF insoluble Arabidopsis root cell walls, EXT1 has 1 Idt, 8 VYK motifs and 14 lone Tyr residues (Figure S1 for protein sequences). Here notably, multiple peptides containing VYK motifs were released by trypsin from WT and ANP10 HFI, in the major peptide fractions (Table 3 and Table 4). This argues against the role of VYK motifs in the formation of covalent cross-links. Indeed, an EXT analog containing six SOOOOTOVYK repeats with no YXY motif was cross-linked very slowly by tomato extensin peroxidase [51] suggesting that VYK might at best be a minor participant in HRGP cross-linking. These data support the argument that EXT1 is held covalently in the protein network mainly through pulcherosine formed between EXT1 and other HRGPs.

EXT3, EXT17 and EXT21 are classical EXTs rich in Idt motifs and lone Tyr residues. EXT3 contains 16 Idt motifs and 15 lone Tyr residues that could be involved in insolubilizing EXT3 by forming Di-Idt and pulcherosine. Pulcherosine may be preferred as suggested by Cannon *et al*. [27] based on the result that it was the major *in vitro* cross-linking product of EXT3 by peroxidase. Much like EXT3, EXT17 contains 24 Idts and 126 lone Tyr residues and EXT21 has 12 Idts and 8 lone Tyr residues, all of which could contribute to their cross-linking in the wall. The high potential of extensive cross-linking of these EXTs might contribute to the observations that their corresponding peptides were only recovered as minor fractions on the peptide maps and the Hyp-rich substances remained in the pellets after protease digestion.

LRX is a family of chimeric EXTs that possess N-terminal non-HRGP Leucine-rich domains and C-terminal EXT domains. They are crucial components of the cross-linked root hair cell wall protein network and contribute to the root hair morphology [52,53,54,55]. LRX proteins were identified previously in the loosely bound cell wall proteome of Arabidopsis cell suspension cultures (LRX2, LRX3 and LRX4) [56] and etiolated hypocotyl walls (LRX3, LRX4 and LRX5) [57]. Here we identified several members of the LRX family proteins in the HFI from ANP and MT walls, confirming that LRXs are firmly bound to cell wall [55]. One common feature of the LRXs identified is that all the peptides isolated were found in the N-terminal non-HRGP Leucine-rich domain and no peptides from the C-terminal EXT domains. This is important since it could be due to the EXT domains of these LRXs being cross-linked in the protein networks. For the extensin domains of the LRX proteins identified, there are 9 Idt motifs and 30 lone Tyr residues in LRX1, 3 Idt motifs and 34 lone Tyr residues in LRX2, 2 Idt motifs and 22 lone Tyr residues in LRX3, and 3 Idt motifs and 18 lone Tyr in LRX5. LRX6 has a relatively small EXT domain, having 5 Idt motifs and 1 lone Tyr residue. One exception is LRX4, which has a very short EXT domain with 5 Tyr residues but no Idt motif. Nevertheless, LRX4 has the potential to form Tyr-related cross-links with other wall HRGPs or through other type of interactions as documented earlier [55]. Similar to LRX4 is HAE2, a short chimeric EXT with 6 lone Tyr residues but no Idt motifs, found in ANP10 HFI. Strikingly, no LRX proteins were found in WT HFI. This could indicate that (1) the LRX are minor components of WT walls, (2) they were extensively cross-linked in the wall and could not be released by trypsin, or (3) the LRX peptides were somehow lost during the peptide collecting process.

Similar to EXTs, PRP3 has 16 VYX (9X = K and 7X = T) motifs and 19 lone Tyr residues while PRP4 has 5 VYK motifs and 8 lone Tyr residues that could potentially insolubilize them in the wall protein network. The mechanism of PRP10 cross-linking in the walls remains unknown since it has no Tyr residues, thus the traditional cross-linking via Tyr does not apply to PRP10.

AGPs have been reported to interact with wall polysaccharides, especially pectin via non-covalent [58] or covalent linkages [8,59]. AGPs also self-associate with each other. The PAC domain of AGP31 was recently reported to interact non-covalently with the polygalactan chains on its Pro-rich domain [60]. Previously, AGP31 was found in etiolated Arabidopsis hypocotyl cell walls [57] and it was also one of the two abundant salt-elutable HRGPs present in Arabidopsis cell suspension cultures [46]. Here we report for the first time AGP peptides isolated from HF deglycosylated walls. For AGP31, due to the removal of sugars on the glycan chain of the Pro-rich domain by HF, the interaction between the PRP-AGP containing Cys (PAC) domain and the Pro-rich domain was disrupted thus a self-association mechanism for the presence of AGP31 in the HFI is unlikely. One possible mechanism for the occurrence of AGP31 could be due to hydrophobic interactions with other wall protein components during the removal of HF. AGP30 has a structure similar to AGP31 with a PAC domain that shares 61% sequence identity with that of AGP31, thus it is likely that these two AGPs were associated with the walls via identical mechanisms.

The FLA proteins are characterized by their fasciclin-like domains that are involved in protein-protein recognition via non-covalent forces, such as hydrophobic interactions [61,62], an interaction that tended to be dissociated by HF treatment [63]. However, upon the removal of HF, these interactions might direct the re-association of FLAs and hold them in the HFI. Other interactions between AGPs or AGP and wall proteins also exist. One example of AGPs covalent cross-linking was reported in sugar beet leaves and this covalent coupling of two AGP molecules was H_2_O_2_ mediated and potentially follows a mechanism similar to the Tyr-involved cross-linking of extensins [64]. This could be one potential mechanism for the cross-linking of the AGPs identified in the wall HFI—through the formation of Tyr derivatives judging by their high Tyr content (Figure S1).

### 3.5. Potential Candidate HRGPs for the WT Phenotype Reversion

EXT3 has unique structural features and functions in Arabidopsis root cell wall by self-assembling into a scaffold that is further rigidified by peroxidase cross-linking [27]. This scaffold formed by EXT3 was proposed [26,27] to serve as a template for the correct deposition of wall polysaccharides that contribute to the cell wall integrity. Structural features of EXT3 include (1) an extended poly-proline II confirmation that ensures maximum contact area between EXT3 molecules during self-assembly and it is contributed by the Ser-(Hyp)_4_ motif along with the arabinosylation on the Hyp residues [65]; (2) the Idt motifs and free Tyr residues ensures the correct alignment of EXT3 molecules in self-assembly and later cross-linking process [27]; (3) a rich content of His and Lys residues enables the ionic interaction between EXT3 and cell wall pectic polysaccharides under physiological conditions [66,67]. Thus to compensate the loss of EXT3 and revise the *EXT3* knockout to wild-type phenotype, the ANPs require an HRGP or a group of HRGPs that possess structural characteristics resemble that of EXT3.

Among the candidate ANP HRGPs for the auto-rescuing of *ext3* knockout phenotype (see Section 2.7), the two LRX proteins (LRX1 and LRX2) both have a C-terminal extensin domain that is similar to EXT3 in protein size and in content of the Ser-(Hyp)_4_ glycomotifs (Figure S1). Both extensin domains also have abundant Tyr-based cross-linking components (elaborated in the previous section). Thus LRX1 and LRX2 could potentially serve as EXT3 substitutes to form a well cross-linked network despite the relative low Lys and His contents in their extensin domains.

PRP4, on the other hand, has a protein size similar to that of EXT3 and its high Lys and His content will enable the ionic interaction with wall polysaccharides (Figure S1). PRP4 also has abundant cross-linking sites to form a covalent protein network (Figure S1). However, PRP4 lacks the Ser-(Hyp)_4_ motifs that reinforce the protein shape (Figure S1). Instead, it contains many repeats of XPPP motif (X = K, H, V, I, Figure S1). The XPPP motifs are sufficient to form a poly-Proine II confirmation with contiguous *trans* polyproline [68] and thus stabilize the protein structure of PRP4. Combining all the structural features, PRP4 also has the potential to replace EXT3 in the cell wall to reverse the WT phenotype.

### 3.6. Gene Expression vs. Protein Identification by Proteomics

Our results serve as a general survey of the HRGPs from WT and *ext3* knockout lines. However, our findings of the wall HRGP members in ANP and MT walls via proteomics approach showed poor correspondence with the members proposed in the self-rescuing model of ANPs by microarray and qRT-PCR [28], with only one HRGP, PRP4, common to both studies. Both approaches should be considered in determining the molecular composition of the cell walls as each has its limitations: HRGPs are structural proteins that stably remain bound in the network after their production, however, not all of them are equally accessible during protein extraction procedures, and the most abundant of them are likely to be the best represented, while the less abundant may be providing a vital wall function, but not detectable. In the case of taking gene expression as an indication of HRGP presence in the wall, it must be considered that the technology is measuring a snap shot of the messages at a particular time, and not necessarily all of that message will be translated or modified to become stable networked HRGPs. Meanwhile, the uncoupling of gene expression and RNA translation, for instance regulated by endogenous double-stranded RNA interference [69] or microRNA [70], could also be responsible for the difference in gene expression and protein abundance.

## 4. Experimental Section

### 4.1. Arabidopsis Seedling Culture

Seeds of wild-type *Arabidopsis* Landsberg *erecta* (WT, purchased from Lehle Seeds, Brushy Creek, TX, USA), ANP lines #4 and #10 (ANP4 and ANP10), and heterozygote *RSH*/*rsh* were surface sterilized before being planted on solid ½ MS medium plates (pH 5.8) containing 8 g/L agar. Seeds of WT and ANP lines were planted along a line 3 cm from the top of the plates and the plates were positioned vertically and cultured for a total of 14 days. *RSH*/rsh heterozygote seeds were scattered onto plates and placed horizontally for 21 days. To synchronize seed germination, the plated seeds were first incubated at 4 °C for two days before maintained at room temperature. All seeded plates were kept under constant fluorescent light.

### 4.2. Root Cell Wall Isolation

Root cell walls were prepared using a protocol by York *et al*. [71]. The roots were homogenized by grinding in liquid N_2_ to a fine powder followed by sonication in 35 mL 0.5 M K-P (K_2_HPO_4_: KH_2_PO_4_ = 2:1 molar ratio) buffer. Cell breakage was evaluated by optical microscopy. The wall fragments were pelleted by centrifugation at 11,000 *g* for 15 min, washed three times with 45 mL 0.5 M K-P buffer followed by four washes with 0.1 M K-P buffer and four washes with cold water to remove cellular contents. The wall pellets were then extracted four times with an extraction solution (chloroform: methanol = 1:1, v/v) to remove membrane components and collected by centrifugation at 20,000 *g* for 15 min after each extraction. The wall pellets were washed with 95% ethanol and water to remove extraction mixture and then lyophilized.

### 4.3. Wall Deglycosylation with HF

Cell wall from WT, ANP, and MT were deglycosylated with anhydrous hydrogen fluoride (HF) using methods described previously [2]. The HF insoluble pellets (HFI) were dialyzed against distilled water to remove excessive HF then collected by centrifugation. The HFIs were re-suspended in water and washed four times before lyophilization.

### 4.4. Trypsin Digestion of HFI

WT and ANP HFI were resuspended in 500 µL of water and incubated at 110 °C for 10 min. The suspensions were cooled and the following reagents were added: 1280 µL water, 100 µLof 1 M NH_4_HCO_3_ (final [NH_4_HCO_3_] = 0.05 M, pH 8.0), 100 µL of 1 mg/mL trypsin (pellet: trypsin = 100:1, w/w) and 20 µL of 1 M CaCl_2_ (final [Ca^2+^] = 10 mM) to make a 2 mL reaction mixture with a HFI concentration at 5 mg/mL. The reactions were incubated at room temperature with constant stirring (200 rpm) for 24 h and another 100 µL of trypsin solution was added for another 24-h incubation. The trypsin insoluble HFI (TI HFI) was collected by centrifugation, washed four times with water and then lyophilized. The supernatant from each digestion and the water washes were pooled, lyophilized and used for peptide isolation.

MT HFI was re-suspended in 100 µL water with 2 µL Tris(2-carboxyethyl) phosphine hydrochloride and incubated at 95 °C for 10 min. The suspension was cooled and pre-treated with 2 µL 0.75 M Iodoacetamide in dark for 1 h and 2 µL 0.25 M DL-Dithiothreitol for 10 min. After pretreatment, the solution was mixed with 47 µL H_2_O, 20 µL 0.5M NH_4_HCO_3_, 20 µL acetonitrile and 7 µL of 1 mg/mL trypsin (Trypsin Gold, Promega) to make a total volume of 200 µL with a HFI concentration at 5 mg/mL and kept at 37 °C with shaking (200 rpm) for 24 h. Seven microliters of trypsin was added after the first 24-h incubation period for another 24-h incubation. The TI HFI was washed and collected as mentioned earlier and the supernatant was lyophilized and sent for proteomic analysis.

### 4.5. Pronase Degradation

TI HFI from WT and ANP were digested with pronase using the trypsin digestions protocol described above. After digestion, the pronase insoluble HFI (PI HFI) were collected by centrifugation, washed four times with water and lyophilized.

### 4.6. Tryptic Peptide Isolation

Typtic peptides were isolated from HFI following the workflow shown in Figure 1. Lyophilized supernatants from trypsin digested WT and ANP HFI were dissolved at 2 mg/mL in 50 mM formic acid and fractionated by a PolyHYDROXYETHYL A column in size exclusion (SEC) mode. The column was eluted isocratically at a flow rate of 0.2 mL/min with the eluate monitored at 220 nm. One-minute fractions were collected and combined into three groups: (1) fractions 7 to 10; (2) fractions 11 and 12; and (3) fractions 13 to 15. Fraction groups were lyophilized, dissolved in loading buffer (10 mM KH_2_PO_4_ with 20% acetonitrile, pH 2.8), and further fractionated on a strong cation exchange (SCX) PolySULFOETHYL A column. The column was eluted at 0.2 mL/min using a 60 min gradient of 0 to 100% wash buffer (10 mM KH_2_PO_4_ with 0.8 M KCl, pH 2.8) followed by 15 min elution with 100% wash buffer. The eluate was monitored at 220 nm and two-minute fractions were collected.

Fractions from SCX were combined in groups (Figure 1), lyophilized, and dissolved in Buffer A (0.1% TFA, v/v) for further fractionation on a reverse-phase PRP-1 column using a 100 min gradient of 0 to 50% Buffer B (0.1% TFA, 80% acetonitrile, v/v) at a flow rate of 0.5 mL/min for WT and ANP10 peptides and a 100-min gradient of 0 to 70% Buffer B for ANP4 peptides. The eluate absorbance was monitored at 220 nm and the eluted peaks were collected and lyophilized before sequencing by MS/MS.

### 4.7. Peptide Sequencing by Mass Spectrometry

Peptide sequencing of isolated peptide peaks (WT and ANP) and the unfractionated MT tryptic digest were performed at the Proteomic Facility at Michigan State University (E. Lansing, MI, USA). Detailed protocols were listed under supplementary material.

## 5. Conclusions

Here we reveal unique HRGP networks in wild-type Arabidopsis and *ext3* knockout lines using peptide isolation and sequencing approaches. Our methodology released wall HRGP components for identification. EXT3 is a major HRGP component in the WT cell wall and our approach did not find EXT3 in any of the ANP walls, confirming that the auto phenotypic auto-revertant ANPs can assemble functional walls by an alternative mechanism to that involving EXT3. Furthermore we found indications of a difference between the walls of WT and ANPs, thereby providing an opportunity to compare the molecular structure and related functional mechanisms of two different cell wall types of apparently similar function.

## Supplementary Files

Supplementary File 1
