# Supplementary material for: Identification of the Abundant Hydroxyproline-Rich Glycoproteins in the Root Walls of Wild-Type Arabidopsis, an ext3 Mutant Line, and Its Phenotypic Revertant
Source: Plants (Basel). 2015 Jan 21;4(1):85–111. doi: 10.3390/plants4010085 (PMC4844335; doi:10.3390/plants4010085)
Supplement: Supplementary File 1 [file plants-04-00085-s001.pdf]

## Supplementary Material

## Material and Methods

*WT and ANP Peptide Sequencing by Mass Spectrometry*

Peptide sequencing by LC/MS/MS was carried out at The Proteomics Core Facility, Michigan State University (East Lansing, MI, USA). Samples were suspended in 2% acetonitrile/0.1% TFA to 25 µL and from this 10 µL were automatically injected (4 µL/min in 2% acetonitrile/0.1% formic acid) by a Waters nanoAcquity Sample Manager onto a Waters Symmetry C18 peptide trap column (5 µm, 180 µm × 20 mm). Bound peptides were eluted from the C18 column onto a Michrom MAGIC C18AQ column (3 µ, 200A, 100U × 150 mm) and the peptides were further eluted over 15 minutes with a gradient of 5% B to 35% B in 8 min (Buffer A = 99.9% Water/0.1% formic acid; Buffer B = 99.9% acetonitrile/0.1% formic acid) on the C18AQ column. The C18AQ column elution gradient was then quickly ramped to 90% B at 9 min, held for 0.5 min and returned to 5% B for the remainder of the run at a constant flow rate of 0.8 µL/min.

Peptides eluted from the C18AQ column were sprayed into a ThermoFisher LTQ-FT Ultra mass spectrometer using a Michrom ADVANCE nanospray source. Survey scans were taken in the FT (25,000 resolution determined at m/z 400) and the top five ions in each survey scan were subjected to automatic low energy collision induced dissociation (CID) in the LTQ. The resulting MS/MS spectra were converted to peak lists using BioWorks Browser v3.3.1 by default LTQ-FT parameters and searched against a custom protein database consisting of Arabidopsis sequences (TAIR v10, downloaded from www.Arabidopsis.org) and common processing contaminants such as trypsin and human keratins (downloaded from NCBI, www.ncbi.nlm.nih.gov), using the Mascot searching algorithm, v 2.3 (www.matrixscience.com). The Mascot output was then analyzed using Scaffold, v3.5.1 (www.proteomesoftware.com) to probabilistically validate protein identifications using the ProteinProphet computer algorithm. Assignments validated above the Scaffold 95% confidence filter were considered true.

*Sequencing of Peptides from MT*

The MT HFI trypsin digest was re-suspended in 2% acetonitrile/0.1% TFA (designated Blank Ssolution) to 25 µL and purified by solid phase extraction using OMIX c18 tips (www.agilent.com) according to the manufacturer's instructions. Eluted peptides were dried by vacuum centrifugation and re-suspended in 20 µL Blank Solution. From this, 5 µL were automatically injected by a Thermo (www.thermo.com) EASYnLC onto a Thermo Acclaim PepMap RSLC 0.075 mm × 150 mm C18 column and eluted over 60 min with a gradient of 5% B to 30% B in 49 min, ramping to 100% B at 50 min and held at 100% B for the duration of the run (Buffer A = 99.9% water/0.1% formic acid; Buffer B = 99.9% acetonitrile/0.1% formic acid).

Eluted peptides were sprayed into a ThermoFisher Q-Exactive mass spectrometer (www.thermo.com) using a FlexSpray spray ion source. Survey scans were taken in the Orbi trap (35,000 resolution, determined at *m/z* 200) and the top twelve ions in each survey scan are then subjected to automatic higher energy collision induced dissociation (HCD) with fragment spectra acquired at 17,500 resolution. The resulting MS/MS spectra are converted to peak lists using Mascot Distiller, v2.4.3.3 (www.matrixscience.com) and searched against all entries in the TAIR, v10, protein sequence database

(downloaded from The Arabidopsis Information Resource, www.arabidopsis.org) appended with common laboratory contaminants (downloaded from www.thegpm.org, cRAP project) and also against all protein entries available from SwissProt (v2013_10, downloaded from www.uniprot.org), using the Mascot searching algorithm, v2.4. The Mascot output was then analyzed using Scaffold, v4.2.1 (www.proteomesoftware.com) to probabilistically validate protein identifications. Assignments validated above the Scaffold 95% confidence filter were considered true.

**Table S1.** Amino acid composition (mole %) of major reverse-phase peptide peaks from the trypsin digested cell wall HF insoluble pellets.

| Amino Acid | W1 | W2 | W3 | W4 | W5 | W6 | W7 | W8 | W9 | W10 | F1 | F2 | F3 | F4 | A1 | A2 | A3 | A4 | A5 | A6 |
|---|---|---|---|---|---|---|---|---|---|---|---|---|---|---|---|---|---|---|---|---|
| Asp | 0 | 0 | 0 | 0 | 0 | 3 | 5 | 6 | 8 | 0 | 0 | 0 | 5 | 8 | 4 | 11 | 5 | 6 | 6 | 10 |
| Glu | 0 | 0 | 0 | 3 | 0 | 3 | 4 | 2 | 0 | 0 | 0 | 0 | 0 | 6 | 2 | 6 | 7 | 4 | 5 | 7 |
| **Hyp** | **23** | **8** | **36** | **18** | **33** | **33** | **23** | **16** | **28** | **33** | **32** | **41** | **28** | **14** | **16** | **3** | **0** | **17** | **18** | **17** |
| Ser | 10 | 1 | 15 | 9 | 15 | 10 | 12 | 13 | 13 | 20 | 18 | 25 | 13 | 10 | 8 | 22 | 6 | 14 | 15 | 11 |
| Gly | 0 | 2 | 5 | 12 | 0 | 7 | 10 | 7 | 0 | 8 | 0 | 8 | 8 | 13 | 7 | 21 | 22 | 13 | 17 | 14 |
| His | 0 | 0 | 20 | 34 | 0 | 0 | 12 | 6 | 0 | 10 | 22 | 0 | 0 | 9 | 0 | 0 | 0 | 15 | 10 | 0 |
| Arg | 0 | 0 | 0 | 0 | 0 | 0 | 5 | 4 | 0 | 0 | 0 | 0 | 0 | 6 | 0 | 0 | 0 | 4 | 4 | 4 |
| Thr | 2 | 2 | 0 | 0 | 0 | 12 | 3 | 1 | 0 | 0 | 0 | 0 | 10 | 0 | 7 | 7 | 0 | 3 | 0 | 7 |
| Ala | 7 | 81 | 11 | 14 | 6 | 6 | 8 | 7 | 51 | 18 | 12 | 0 | 8 | 0 | 39 | 0 | 23 | 0 | 0 | 0 |
| Pro | 0 | 1 | 2 | 0 | 0 | 2 | 3 | 4 | 0 | 0 | 0 | 0 | 0 | 2 | 2 | 8 | 9 | 3 | 4 | 5 |
| Tyr | 0 | 0 | 0 | 0 | 0 | 0 | 0 | 14 | 0 | 12 | 0 | 0 | 0 | 2 | 0 | 0 | 0 | 3 | 0 | 0 |
| Val | 2 | 0 | 0 | 0 | 3 | 0 | 4 | 2 | 0 | 0 | 0 | 0 | 0 | 3 | 2 | 6 | 3 | 3 | 5 | 4 |
| Ile | 6 | 0 | 0 | 0 | 5 | 0 | 0 | 5 | 0 | 0 | 0 | 0 | 0 | 5 | 0 | 0 | 6 | 0 | 0 | 0 |
| Leu | 34 | 0 | 0 | 0 | 25 | 0 | 0 | 7 | 0 | 0 | 0 | 0 | 0 | 7 | 0 | 0 | 11 | 0 | 0 | 0 |
| Phe | 0 | 0 | 0 | 0 | 0 | 5 | 0 | 0 | 0 | 0 | 0 | 0 | 6 | 0 | 0 | 0 | 0 | 0 | 0 | 0 |
| Lys | 17 | 5 | 12 | 10 | 15 | 19 | 10 | 5 | 0 | 0 | 15 | 25 | 22 | 16 | 14 | 16 | 9 | 14 | 15 | 20 |

1. Zero value indicated a low signal during analysis that could not be integrated by the analyzer. 2. The amino acid composition of F5 and A7 were not determined due to the lack of material.

**EXT1 (AT1G76930):**

*MGAPMASFLV LAFSLAFVSQ TTA*NYFYSSP PPPVKHYSPP PVYKSPPPPV K**HYSPPPVYK**
SPPPPVKHYS PPPVYKSPPP PVK**YYSPPPV YK**SPPPPVYK SPPPPVKHYS PPPVYKSPPP
PVKHYSPPPV YKSPPPPVKH YSPPPVYKSP PPPVKHYSPP PSYTTLHHHR FTTHLLQSYT
TLHHHRFTTH LLQLYTTPHH HPRSTTNTNL LLLRSITLPL RYTILLLLQY TTTHLHTNPT
FTNHHLLLTT RLLNRL

**EXT3 (AT1G21310):**

*MGSPMASLVA TLLVLTISLT FVSQSTA*NYF YSSPPPPVKH YTPPVKHYSP PPVYHSPPPP
KKHYEYKSPP PPVKHYSPPP VYHSPPPPKK HYVYKSPPPP VKHYSPPPVY HSPPPPKKHY
VYKSPPPPVK HYSPPPVYHS PPPPKKHYVY KSPPPPVKHY SPPPVYHSPP PPKKHYVYKS
PPPPVKHYSP PPVYHSPPPP KKHYVYKSPP PPVKHYSPPP VYHSPPPPKK HYVYKSPPPP
VKHYSPPPVY HSPPPPKKHY VYKSPPPPVK HYSPPPVYHS PPPPKKHYVY KSPPPPVKHY
SPPPVYHSPP PPKKHYVYKS PPPPVKHYSP PPVYHSPPPP KKHYVYKSPP PPVKHYSPPP
VYHSPPPPKK HYVYKSPPPP VKHYSPPPVY HSPPPPKEKY VYKSPPPPPV HHYSPPHHPY
LYKSPPPPYH Y

**EXT17 (AT3G54580):**

*MGPSTHLICA LGVVIMATMV AA*YDPYTDSS PPPLYSSPLP KIEYKTPPLP YIDSSPPPTY
SPAPEVEYKS PPPPYVYSSP PPPTYSPSPK VEYKSPPPPY VYSSPPPPTY SPSPKVEYKS
PPPPYVYSSP PPPTYSPSPK VEYKSPPPPY VYSSPPPPTY SPSPKVEYKS PPPPYVYSSP
PPPTYSPSPK VEYKSPPPPY VYSSPPPPYY SPSPKVEYKS PPPPYVYSSP PPPTYSPSPK
VDYKSPPPPY VYSSPPPPYY SPSPKVEYKS PPPPYVYSSP PPPTYSPSPK VDYKSPPPPY
VYSSPPPPYY SPSPKVDYKS PPPPYVYSSP PPPTYSPSPK VEYKSPPPPY VYSSPPPPTY
SPSPKVEYKS PPPPYVYSSP PPPTYSPSPK VEYKSPPPPY VYSSPPPPTY SPSPKVEYKS
PPPPYVYSSP PPPYYSPSPK VEYKSPPPPY VYSSPPPPTY SPSPKVDYKS PPPPYVYSSP
PPPYYSPSPK VYYKSPPPPY VYSSPPPPYY SPSPKVYYKS PPPPYVYSSP PPPYYSPSPK
VYYKSPPPPY YSPSPKVYYK SPPPPYVYSS PPPPYYSPSP KVYYKSPPPP YVYSSPPPPY
HSPSPKVQYK SPPPPYVYSS PPPPYYSPSP KVYYKSPPPP YVYSSPPPPY YSPSPKVYYK
SPPPPYVYSS PPPPYYSPSP KVYYKSPPHP HVCVCPPPPP CYSPSPKVVY KSPPPPYVYS
SPPPPHYSPS PKVYYKSPPP PYVYSSPPPP YYSPSPKVHY KSPPPPYYAP TPKVHYKSPP
PPYVYSSPPP PYYSPSPKVH YKSPPPPYVY SSPPPPYYSP SPKVEYKSPP PPYVYSSPPP
PYYSPSPKVD YKSPPPPYVY SSPPPPYYSP SPKVDYKSPP PPYVYSSPPP PYYSPSPVVD
YKSPPPPYVY SSPPPPYYSP SPKVEYKSPP PPYVYKSPPP PSYSPSPKTE Y

**EXT21 (AT2G43150)**:

*MATPAWSHAK AQWVVAMLAL LVGSAMAT*EP YYYSSPPPPY EYKSPPPPVK SPPPPYEYKS
PPPPVKSPPP PYYYHSPPPP VKSPPPPYVY SSPPPPVKSP PPPYYYHSPP PPVKSPPPPY
YYHSPPPPVK SPPPPYYYHS PPPPVKSPPP PYYYHSPPPP VKSPPPPYYY HSPPPPVKSP
PPPYLYSSPP PPVKSPPPPV YIYASPPPPT HY

**Figure S1.** *Cont.*

**LRX1 (AT1G12040):**

*MLFPPLRSLF LFTLLLSSVC FLQIKAD*HDD ESDLGSDIKV DKRLKFENPK LRQAYIALQS
WKKAIFSDPF NFTANWNGSD VCSYNGIYCA PSPSYPKTRV VAGIDLNHAD MAGYLASELG
LLSDLALFHI NSNRFCGEVP LTFNRMKLLY ELDLSNNRFV GKFPKVVLSL PSLKFLDLRY
NEFEGKIPSK LFDRELDAIF LNHNRFRFGI PKNMGNSPVS ALVLADNNLG GCIPGSIGQM
GKTLNELILS NDNLTGCLPP QIGNLKKVTV FDITSNRLQG PLPSSVGNMK SLEELHVANN
AFTGVIPPSI CQLSNLENFT YSSNYFSGRP PICAASLLAD IVVNGTMNCI TGLARQRSDK
QCSSLLARPV DCSKFGCYNI FSPPPPTFKM SPEVRTLPPP IYVYSSPPPP PSSKMSPTVR
AYSPPPPPSS KMSPSVRAYS PPPPPYSKMS PSVRAYPPPP PPSPSPPPPY VYSSPPPPYV
YSSPPPPPYV YSSPPPPPYV YSSPPPPYVY SSPPPPYVYS SPPPPPPSPP PPCPESSPPP
PVVYYAPVTQ SPPPPSPVYY PPVTQSPPPP SPVYYPPVTN SPPPPSPVYY PPVTYSPPPP
SPVYYPQVTP SPPPPSPLYY PPVTPSPPPP SPVYYPPVTP SPPPPSPVYY PPVTPSPPPP
SPVYYPSETQ SPPPPTEYYY SPSQSPPPTK ACKEGHPPQA TPSYEPPPEY SYSSSPPPPS
PTSYFPPMPS VSYDASPPPP PSYY

**LRX2 (AT1G62440):**

MESSDDCFIQ LEVSVRLGVN DLLSFENYRT SFPWDSYSGL DGFFHLGAVD DEGNLTEIGQ
LMNLEIRGDD DDDDISDDNI KVDPSLKFEN PSLRQAYIAL QSWKQAIFSD PFNFTANWNG
SDVCSYNGIF CAPSPSSPKT RVVAGIDLNH ADMAGYLPRE LGLLTDLALF HLNSNRFCGE
VPLTFKHMKL LFELDLSNNR FVGKFPNVVL SLPSLKFLDL RYNEFEGSIP SKLFDKELDA
IFLNHNRFMF GIPENMGNSP VSALVLADND LGGCIPGSIG LMGKTLNEII LSNDNLTGCL
PPQIGNLKNV TVFDISFNRL SGPLPSSIGN MKSLEQLNVA NNRFTGVIPS SICQLSNLEN
FTYSSNFFTG DAPRCVALLG DNVVVNGSMN CIDGKEDQRS SKECSSPASR SVDCSKFGCN
NFFSPPPPSF KMSPTVRVLP PPPPSSKMSP TFRATPPPPS SKMSPSFRAT PPPPSSKMSP
SFRATPPPPS SKMSPSVKAY PPPPPPPEYE PSPPPPSSEM SPSVRAYPPP PPLSPPPPSP
PPPYIYSSPP PPSPSPPPPY IYSSPPPVVN CPPTTQSPPP PKYEQTPSPR EYYPSPSPPY
YQYTSSPPPP TYYATQSPPP PPPPTYYAVQ SPPPPPPVYY PPVTASPPPP PVYYTPVIQS
PPPPPVYYSP VTQSPPPPPP VYYPPVTQSP PPSPVYYPPV TQSPPPPPVY YLPVTQSPPP
PSPVYYPPVA KSPPPPSPVY YPPVTQSPPP PSTPVEYHPP ASPNQSPPPE YQSPPPKGCN
DSPSNDHHYQ TPTPPSLPPP YYEDTPLPPI RGVSYASPPP PSIPYY

**LRX3 (AT4G13340):**

*MKKTIQILLF FFFLINLTNA* LSISSDGGVL SDNEVRHIQR RQLLEFAERS VKITVDPSLN
FENPRLRNAY IALQAWKQAI LSDPNNFTSN WIGSNVCNYT GVFCSPALDN RKIRTVAGID
LNHADIAGYL PEELGLLSDL ALFHVNSNRF CGTVPHRFNR LKLLFELDLS NNRFAGKFPT
VVLQLPSLKF LDLRFNEFEG TVPKELFSKD LDAIFINHNR FRFELPENFG DSPVSVIVLA
NNRFHGCVPS SLVEMKNLNE IIFMNNGLNS CLPSDIGRLK NVTVFDVSFN ELVGPLPESV
GEMVSVEQLN VAHNMLSGKI PASICQLPKL ENFTYSYNFF TGEAPVCLRL PEFDDRRNCL
PGRPAQRSPG QCKAFLSRPP VNCGSFSCGR SVSPRPPVVT PLPPPSLPSP PPPAPIFSTP
PTLTSPPPPS PPPPVYSPPP PPPPPPPVYS PPPPPPPPPP PPVYSPPPPP PPPPPPPPVY
SPPPPSPPPP PPPVYSPPPP PPPPPPPPVY SPPPPPVYSS PPPPPSPAPT PVYCTRPPPP
PPHSPPPPQF SPPPPEPYYY SSPPPPHSSP PPHSPPPPHS PPPPIYPYLS PPPPPTPVSS
PPPTPVYSPP PPPPCIEPPP PPPCIEYSPP PPPPVVHYSS PPPPPVYYSS PPPPPVYYSS
PPPPPPVHYS SPPPPEVHYH SPPPSPVHYS SPPPPPSAPC EESPPPAPVV HHSPPPPMVH
HSPPPPVIHQ SPPPPSPEYE GPLPPVIGVS YASPPPPPFY

**Figure S1.** *Cont.*

**LRX4 (AT3G24480):**

MKNNTTQSLL LLLLFFFFFF EISHSLSISS NAPLSDTEVR FIQRRQLLYY RDEFGDRGEN
VTVDPSLIFE NPRLRSAYIA LQAWKQAILS DPNNITVNWI GSNVCNYTGV FCSKALDNRK
IRTVAGIDLN HADIAGYLPE ELGLLTDLAL FHVNSNRFCG TVPHKFKQLK LLFELDLSNN
RFAGKFPTVV LHLPSLKFLD LRFNEFEGTV PKELFSKNLD AIFINHNRFR FELPENFGDS
PVSVIVLANN HFHGCIPTSL VEMKNLNEII FMNNGLNSCL PADIGRLKNV TVFDVSFNEL
VGPLPESVGG MVEVEQLNVA HNLLSGKIPA SICQLPKLEN FTYSYNFFTG EAPVCLRLSE
FDDRRNCLPG RPAQRSSRQC SAFLSRPSVD CGSFGCGRSV VKPSPPIVAL PPPPPPSPPL
PPPVYSPPPS PPVFSPPPSP PVYSPPPPPS IHYSSPPPPP VHHSSPPPPS PEFEGPLPPV
IGVSYASPPP PPFY

**LRX5 (AT4G18670):**

MKTKMMMKNT SLIFVLLFIT FFFTSISYSL SLTFNGDLSD NEVRLITQRQ LLYFRDEFGD
RGENVDVDPS LVFENPRLRN AYIALQAWKQ AILSDPNNFT TNWIGSDVCS YTGVYCAPAL
DNRRIRTVAG IDLNHADIAG YLPQELGLLT DLALFHINSN RFCGTVPHRF NRLKLLFELD
LSNNRFAGIF PTVVLQLPSL KFLDLRFNEF EGPVPRELFS KDLDAIFINH NRFRFELPDN
LGDSPVSVIV VANNHFHGCI PTSLGDMRNL EEIIFMENGF NSCLPSQIGR LKNVTVFDFS
FNELVGSLPA SIGGMVSMEQ LNVAHNRFSG KIPATICQLP RLENFTFSYN FFTGEPPVCL
GLPGFDDRRN CLPARPAQRS PGQCAAFSSL PPVDCGSFGC GRSTRPPVVV PSPPTTPSPG
GSPPSPSISP SPPITVPSPP TTPSPGGSPP SPSIVPSPPS TTPSPGSPPT SPTTPTPGGS
PPSSPTTPTP GGSPPSSPTT PTPGGSPPSS PTTPSPGGSP PSPSISPSPP ITVPSPPSTP
TSPGSPPSPS SPTPSSPIPS PPTPSTPPTP ISPGQNSPPI IPSPPFTGPS PPSSPSPPLP
PVIPSPPIVG PTPSSPPPST PTPVYSPPPP STGYPPPPPF TGYSPPSPPP PPPPTFSPSP
SIPPPPPQTY SPFPPPPPPP PQTYYPPQPS PSQPPQSPIY GTPPPSPIPY LPSPPQFASP
PPPAPYYYSS PQPPPPPHYS LPPPTPTYHY ISPPPPPTPI HSPPPQSHPP CIEYSPPPPP
TVHYNPPPPP SPAHYSPPPS PPVYYYNSPP PPPAVHYSPP PPPVIHHSQP PPPPIYEGPL
PPIPGISYAS PPPPPFY

**LRX6 (AT3G22800):**

MREDTFFFQW WFLVSGLSFI FLLPQAFTYH TPPINPCFAH PFLPPITNPR LLKAFTALQA
WKFTITSDPN GFTSNWCGPN VCNYTGVFCA PALDNPYVLT VAGIDLNHAN IAGYLPLELG
LLTDLALFHI NSNRFQGQLP KTLKCLHLLH ELDVSNNKLS GEFPSVIFSL PSLKFLDIRF
NEFQGDVPSQ LFDLNLDALF INDNKFQFRL PRNIGNSPVS VLVLANNDLQ GSCVPPSFYK
MGKTLHEIII TNSQLTGCLN REIGLLNQLT VFDVSYNNLV GSLPETIGDM KSLEQLNIAH
NKFSGYIPES ICRLPRLENF TYSYNFFSGE PPACLRLQEF DDRRNCLPSR PMQRSLAECK
SFSSYPIDCA SFGCSPPSPP PPPPPPPPPP PPPPPPPPPP PPPPPPPYVY PSPPPPPPSP
PPYVYPPPPP PYVYPPPPSP PYVYPPPPPS PQPYMYPSPP CNDLPTPVHY

**HAE2 (AT3G50580):**

MKTSIVLVAA AFLCLVAFPT TTVGKYWPKI EGWPNPSEIT RNELMLLNTG HSFGYGDSKV
WKCTYSNGSA PAISISPSTP IPSTPSTPSP PPPAPKKSPP PPTPKKSPSP PSLTPFVPHP
TPKKSPSPPP TPSLPPPAPK KSPSTPSLPP PTPKKSPPPP PSHHSSSPSN PPHHQQNPWE
HIERCMINMG PVGMCRMQME VSFYTRLFQV SDYCCNLVVN MKSECDDVAW GFFNDPFFVP
LVRYTCHVTC

**Figure S1.** *Cont.*

**AGP30 (AT2G33790):**

*MGIIGKSVSL TLFALLCFTS SVFT*LGVNQP GSSDPFHSLP QHLPLPPIKL PTLPPAKAPI
KLPAYPPAKA PIKLPTLPPA KAPIKLPTLP PIKPPVLPPV YPPKYNKTLV AVRGVVYCKA
CKYAGVNNVQ GAKPVKDAVV RLVCKNKKNS ISETKTDKNG YFMLLAPKTV TNYDIKGCRA
FLVKSPDTKC SKVSSLHDGG KGSVLKPVLK PGFSSTIMRW FKYSVYNVGP FAFEPTCPK

**AGP 31 (AT1G28290):**

*MGFIGKSVLV SLVALWCFTS SVFT*EEVNHK TQTPSLAPAP APYHHGHHHP HPPHHHHPHP
HPHPHPPAKS PVKPPVKAPV SPPAKPPVKP PVYPPTKAPV KPPTKPPVKP PVSPPAKPPV
KPPVYPPTKA PVKPPTKPPV KPPVYPPTKP PVYPPTKAPV KPPVSPPTKP PVTPPVYPPK
FNRSLVAVRG TVYCKSCKYA AFNTLLGAKP IEGATVKLVC KSKKNITAET TTDKNGYFLL
LAPKTVTNFG FRGCRVYLVK SKDYKCSKVS KLFGGDVGAE LKPEKKLGKS TVVVNKLVYG
LFNVGPFAFN PSCPK

**FLA7 (AT2G04780):**

*MAKMQLSIFI AVVALIVCSA SA*KTASPPAP VLPPTPAPAP APENVNLTEL LSVAGPFHTF
LDYLLSTGVI ETFQNQANNT EEGITIFVPK DDAFKAQKNP PLSNLTKDQL KQLVLFHALP
HYYSLSEFKN LSQSGPVSTF AGGQYSLKFT DVSGTVRIDS LWTRTKVSSS VFSTDPVAVY
QVNRVLLPEA IFGTDVPPMP APAPAPIVSA PSDSPSVADS EGASSPKSSH KNSGQKLLLA
PISMVISGLV ALFL

**FLA15 (AT3G52370):**

*MDDLSKLLFF LLLTISITTA* LPDKPGSGQI NSNSVLVALL DSHYTELAEL VEKALLLQTL
EEAVGQHNIT IFAPRNDALE KNLDPEFKSF LLQPKNLKSL QSLLMFHILP KRITSPQFSS
AVVSHRTLSN DHLHFTNGKV NSAEITKPDD LTRPDGIIHG IERLLIPRSV QEDFNRRRSL
RSIAAVLPEG APEVDPRTHR LKKKPAPIPA GAPPVLPVYD AMSPGPSLAP APAPGPGGPR
HHFNGEAQVK DFIHTLLHYG GYNEMADILV NLTSLATEMG RLVSEGYVLT VLAPNDEAMA
KLTTDQLSEP GAPEQIMYYH IIPEYQTEES MYNSVRRFGK IRYDSLRFPH KVEAQEADGS
VKFGHGDGSA YLFDPDIYTD GRISVQGIDG VLFPEEKTPV EKKTGVPVVK KAPKPRRGKL
MEVACTMLGS QFPTCQ

**FLA16 (AT2G35860):**

*MDSSYGATKF LLLLFLTTSI ATA*LPDNKPV PGQINSNSVL VALLDSHYTE LAELVEKALL
LQTLEEAVGK HNITIFAPRN DALERNLDPL FKSFLLEPRN LKSLQSLLMF HILPKRITSP
QWPSLSHHHR TLSNDHLHLT VDVNTLKVDS AEIIRPDDVI RPDGIIHGIE RLLIPRSVQE
DFNRRRSLRS ISAVIPEGAP EVDPRTHRLK KPSPAVPAGA PPVLPIYDAM SPGPSLAPAP
APGPGGPRGH FNGDAQVKDF IHTLLHYGGY NEMADILVNL TSLATEMGRL VSEGYVLTVL
APNDEAMAKL TTDQLSEPGA PEQIMYYHII PEYQTEESMY NAVRRFGKVK YDSLRFPHKV
LAQEADGSVK FGHGDGSAYL FDPDIYTDGR ISVQGIDGVL FPKEETPATE IKPAAPVVKK
VSKSRRGKLM EVACRMMGSR FIPCQ

**Figure S1.** *Cont.*

**PAG2 (AT2G25060):**

*MFLSASMASS SLHVAIFSLI FLFSLAAA*NE VTVGGKSGDW KIPPSSSYSF TEWAQKARFK VGDFIVFRYE SGKDSVLEVT KEAYNSCNTT NPLANYTDGE TKVKLDRSGP FYFISGANGH CEKGQKLSLV VISPRHSVIS PAPSPVEFED GPALAPAPIS GSVRLGGCYV VLGLVLGLCA WF

**PRP3 (AT3G62680):**

*MAITRSSLAI CLILSLVTIT TA*DYYSPSSP PVYKSPEHKP TLPSPVYTPP VYKPTLSPPV YTKPTIPPPV YTPPVYKHTP SPPVYTKPTI PPPVYTPPVY KPTLSPPVYT KPTIPPPVYT PPVYKPTPVY TKPTIPPPVY TPPVYKPTPS PPVYKKSPSY SSPPPPYVPK PTYTPTTKPY VPEILKAVDG IILCKNGYET YPILGAKIQI VCSDPASYGK SNTEVVIYSN PTDSKGYFHL SLTSIKDLAY CRVKLYLSPV ETCKNPTNVN KGLTGVPLAL YGYRFYPDKN LELFSVGPFY YTGPKAAPAT PKY

**PRP4 (AT4G38770):**

*MRILPEPRGS VPCLLLLVSV LLSATLSLA*R VVEVVGYAES KIKTPHAFSG LRVTIDCKVN KGHFVTKGSG NIDDKGKFGL NIPHDIVSDN GALKEECYAQ LHSAAGTPCP AHDGLESTKI VFLSKSGDKH ILGLKQNLKF SPEICVSKFF WPMPKLPPFK GFDHPFPLPP PLELPPFLKK PCPPKYSPPV EVPPPVPVYE PPPKKEIPPP VPVYDPPPKK EVPPPVPVYK PPPKVELPPP IPKKPCPPKP PKIEHPPPVP VYKPPPKIEH PPPVPVYKPP PKIEHPPPVP VHKPPKKPCP PKKVDPPPVP VHKPPTKKPC PPKKVDPPPV PVHKPPPKIV IPPPKIEHPP PVPVYKPPPK IEHPPIYIPP IVKKPCPPPV PIYKPPVVIP KKPCPPPVPV YKPPVVVIPK KPCPPLPQLP PLPKFPPLPP KYIHHPKFGK WPPLPPHP

**PRP10 (At5G09530):**

MALMKKSLSA ALLSSPLLII CLIALLADPF SVGARRLLED PKPEIPKLPE LPKFEVPKLP EFPKPELPKL PEFPKPELPK IPEIPKPELP KVPEIPKPEE TKLPDIPKLE LPKFPEIPKP ELPKMPEIPK PELPKVPEIQ KPELPKMPEI PKPELPKFPE IPKPDLPKFP ENSKPEVPKL METEKPEAPK VPEIPKPELP KLPEVPKLEA PKVPEIQKPE LPKMPELPKM PEIQKPELPK LPEVPKLEAP KVPEIQKPEL PKMPELPKMP EIQKPELPKM PEIQKPELPK VPEVPKPELP TVPEVPKSEA PKFPEIPKPE LPKIPEVPKP ELPKVPEITK PAVPEIPKPE LPTMPQLPKL PEFPKVPGTP

**Figure S1.** Protein sequences of HRGPs identified in wild type and AtEXT3 mutant lines. Color-codes of peptides indicate the Arabidopsis lines from which were they identified: WT only—green, ANP only—blue, MT only—red, WT+ANP—underlined, WT+MT—purple, ANP+MT—orange, All—**BOLD**, signal peptides—*italic dark red*.

© 2015 by the authors; licensee MDPI, Basel, Switzerland. This article is an open access article distributed under the terms and conditions of the Creative Commons Attribution license (http://creativecommons.org/licenses/by/4.0/).
